# The effects of exercise type and elbow angle on vertical ground reaction force and muscle activity during a push-up plus exercise

**DOI:** 10.1186/s12891-015-0486-5

**Published:** 2015-02-10

**Authors:** Jun G San Juan, David N Suprak, Sean M Roach, Marc Lyda

**Affiliations:** Department of Physical Education, Health and Recreation, Western Washington University, Bellingham, WA USA; Western Institute of Neuromechanics, Eugene, OR USA

**Keywords:** Scapula, Rehabilitation, Muscle recruitment, Shoulder

## Abstract

**Background:**

Proper alignment of the scapula during upper extremity motion is important in maintaining shoulder joint function and health. Push-up plus exercise is considered as one of the best exercise to strengthen the muscles that stabilize the scapula. The purpose of the study is to examine the effects of push-up plus variants and elbow position on vertical ground reaction force and electromyographical activity of four shoulder muscles during concentric contraction.

**Methods:**

A total of 22 healthy subjects volunteered for the study. Each of the subjects performed both modified and traditional push-up plus. Modified push-up plus was performed with both knees and hands touching the ground while the traditional push-up plus was executed with hands and feet contacting the ground. Electromyography (EMG) of the upper trapezius (UT), lower trapezius (LT), infraspinatus (INFRA), and serratus anterior (SA), and vertical ground reaction forces (vGRF) were collected.

**Results:**

The traditional push-up plus exhibited higher EMG activity in all muscles tested (*P* < .05) and vertical ground reaction force (*P* < .001) compared to modified push-up plus. The highest difference in EMG activity between the two exercises was observed with the Serratus Anterior muscle (22%). Additionally, the traditional push-up plus presented a higher vGRF compared to the modified push-up plus (*P* < .001) by 17%. The SA had the greatest EMG activity compared to the other muscles tested during the concentric phase of the traditional push-up plus, and this did not occur during the plus phase of the exercise.

**Conclusion:**

The highest activity of the serratus anterior occurred at 55° of elbow extension during the concentric phase of the traditional PUP and not at the plus phase of the exercise. This suggests that when prescribing an exercise to target the serratus anterior, a traditional push-up is adequate and the plus-phase is not necessary. However, for patients that cannot perform a traditional push-up, the modified push-up plus would be a great alternative to strengthen their serratus anterior.

## Background

Proper positioning of the scapula during upper extremity movement is crucial for the maintenance of joint function and health, optimal muscle lengths, force production, and bony and soft tissue alignment [1]. This positioning is afforded by the coordinated actions of muscles that both anchor the scapula to the trunk, and work to rotate it in three dimensions during dynamic movements. Substantial research has focused on the normal motions of the scapula and corresponding actions of healthy stabilizing muscles during movement, as well as how these characteristics differ with acute and chronic injury [2-4]. In a healthy shoulder, overhead motion involves rotation of the scapula in such a way that soft tissues in the subacromial space are not impinged upon. In the case of many acute and chronic injuries, authors have consistently reported positioning of the scapula that increases the impingement of soft tissues, and results in pain [5-7]. For this reason, one of the chief objectives in the rehabilitation of shoulder injuries is re-establishing proper scapular positioning during movement. This is often done using several variants of the push-up exercise [8]. The push-up plus (PUP) exercise, a commonly prescribed push-up variant to target scapular muscle stabilizers, is characterized by a standard push-up motion with a protraction of the scapula upon completion of the push-up repetition [8]. Additionally, the PUP has been shown to result in substantial activation of the scapular stabilizer muscle [9].

Many authors have reported that the forces encountered and the activation levels of muscles primarily involved in many push-up variants increase with the intensity of the push-up exercise (i.e., on knees, traditional push-up, feet on exercise ball, slings) [10-13], while the activation of the scapular stabilizing muscles seems to depend on a combination of the weight-bearing demand and degree of arm elevation during the movement [8,14,15]. Both traditional and modified push-up plus variants have been shown to elicit greater activation of the serratus anterior muscle compared to that of the upper trapezius. In fact, Decker et al. [14] reported that SA activation in the modified push-up plus was equivalent to that in the traditional variant, with a lower arm elevation and, even with the lower weight-bearing demand [14,16]. However, the wall push-up elicited a high upper trapezius/serratus anterior ratio [17]. The literature suggests that proper activation of the stabilizing musculature is a major contributor to optimal performance during upper extremity movements, and helps to prevent excessive stresses on associated soft tissues [18]. Therefore, prevention and treatment of shoulder injuries has traditionally included the prescription of many variations of the push-up exercise [14,19]. Although a substantial body of research is available regarding muscle activation levels during various push-up exercises, these studies have used only static postures or differences in the overall muscle activity, and have not incorporated measures of the forces required, in order to support and accelerate the body weight [13,15,20-24]. Similarly, studies examining force output during these variants have focused either on peak and/or average forces across the range of motion (ROM) during a dynamic motion [25,26] or forces exerted at discrete positions within the ROM [16]. These are two areas that need further study, given that muscle lengths and external torques are altered throughout the push-up ROM, leading to changes in scapular kinematics [27]. As a result, the purpose of this study was to examine the effect of push-up plus variant type (traditional vs. modified) and elbow position (5° increments across the range of motion from 100° to full extension) on electromyographical activity of four stabilizer muscles (upper trapezius, lower trapezius, serratus anterior, and infraspinatus) and vertical ground reaction force during concentric phase of the motion. We hypothesized that the traditional PUP would result in higher EMG activity and vertical ground reaction forces compared to the modified PUP.

## Methods

### Participants

**Figure 1:**
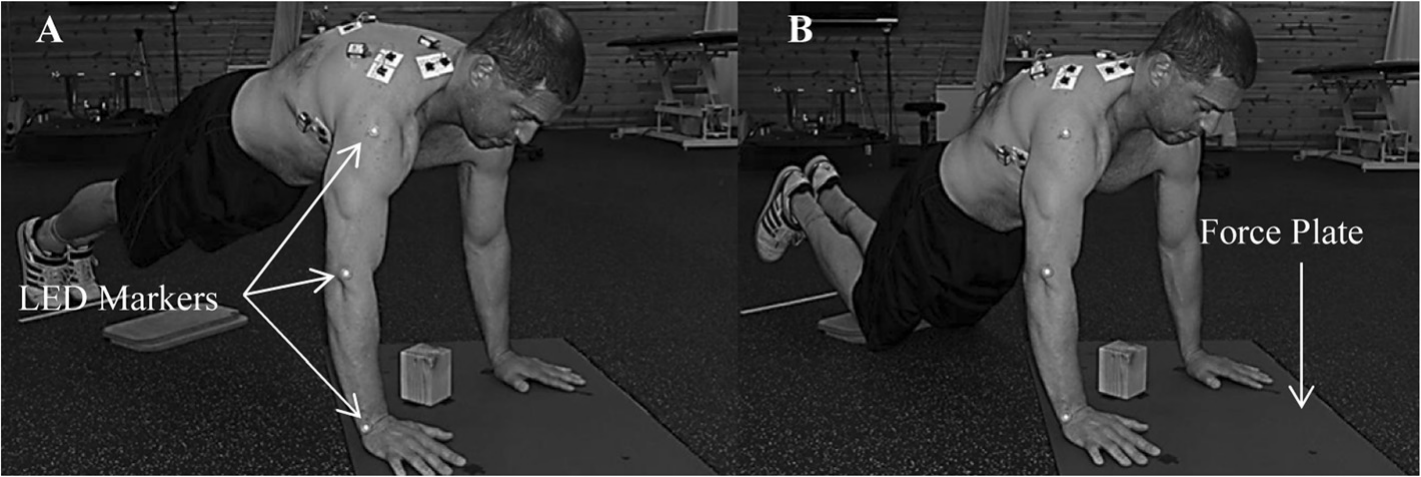
**Push-up plus exercise during (A) Traditional and (B) Modified.**

A total of 22 healthy subjects, 18 males and 4 females, (28.4 ± 10.1 y/o, 176.9 ± 7.9 cm, 75.3 ± 10.3 kg) volunteered for the study. All of the subjects had previous experience in performing the traditional push-up and resistance training. Subjects were included only if they have no history of injury to the shoulder requiring surgery or rehabilitation and if they did not have any documented neurological disorders. The research study was approved by the Western Washington University institutional review board. Each subject signed a consent form and the rights of the subjects were protected. In addition, consent for use of images for publication was obtained from each subject (Figure 1).

All testing was completed in a single session and performed on the dominant upper extremity. The dominant upper extremity was defined as the arm that the subject will use to throw a ball. Subjects performed a standardized warm-up procedure including Codman’s pendulums and stretches for the rotator cuff muscles on both arms. Codman’s pendulum exercises were performed with subjects bent over with the non-dominant hand on a table, and holding a 1.13 kg weight in their dominant hand, letting the weight hang down at arm’s length. Subjects performed one set of 15 repetitions of arm circles, both clockwise and counterclockwise, followed by one set of 15 repetitions of a back and forth movement in the sagittal plane. Stretches consisted of holding a static external and then internal rotation position, both with the shoulder abducted to approximately 90°, for two sets of 15 seconds each.

### Kinematic and kinetic data

The kinematic measurement (Simi Motion, Unterschleissheim, Germany) was utilized to track two-dimensional elbow kinematics across the range of motion of the exercise (Figure 1). A single high speed camera (Basler, Ahrensburg, Germany) set at 120 Hz, was used to collect elbow kinematics. This system consists of tracking active markers that were placed on the lateral acromion, lateral epicondyle and ulnar styloid process. The camera was positioned 3.8 m away from the subject at an angle of approximately 55° from parallel to each subject’s body so that the field of view would remain perpendicular the arm throughout the ROM [27]. Vertical ground reaction force (vGRF) was measured using a force plate (Bertec, Columbus, OH) across the range of motion during execution of each exercise. vGRF data were collected at a frequency of 1000 Hz.

### Electromyography

The Telemyo DTS telemetry (Noraxon, Scottsdale, AZ) EMG system was used to collect muscle activity data. EMG data were collected at 1500 Hz. The unit provided signal amplification, band pass filtering (10 – 1000 Hz), common mode rejection ratio of 100 dB and a final gain of 500. All data were ECG reduced, full-wave rectified, and smoothed using root mean square (10 ms window) through MyoResearch XP Master Ed 1.08 software (Noraxon, Scottsdale, AZ). Disposable Trace 1 (NIKOMED, Huntington Valley, PA) Ag/AgCl surface electrodes were placed on the subject’s dominant arm over the upper trapezius, infraspinatus, lower trapezius and serratus anterior along their primary muscle fiber directions (Figure 1). All muscle locations were determined based on the recommendations by Cram et al. [28]. The pair of electrodes were positioned so that the edges were touching with an inter-electrode distance of 2.5 cm [14]. Before the electrodes were placed on the subject, an alcohol wipe pad was used to clean the skin to help reduce skin impedance.

### Experimental procedures

Kinematic measurements, EMG and ground reaction force data were all internally synchronized. Maximum voluntary isometric contractions (MVC) were collected to normalize EMG amplitude using previously documented procedures [29]. All MVC was done with the subject in a seated position. For the upper trapezius, subjects were asked to position their arm at 90° of abduction and elbow flexed at 90° while the investigator resisted forceful shoulder abduction with a hand positioned at the elbow. For the infraspinatus, subjects were asked to flex their elbow at 90° while arm at the side. A towel was positioned between the elbow and the side of the body to prevent abduction during maximal exertion. Then the subject was instructed to maximally externally rotate the shoulder while resistance was applied at the wrist. For the lower trapezius, a combination of adduction and extension of the shoulder was resisted by the investigator on the elbow while the elbow was flexed at 90° and the shoulder was elevated at 20°. For the serratus anterior, the subject was positioned at 90° of shoulder and elbow flexion and 90° of shoulder internal rotation. The subject was then asked to horizontally adduct the shoulder while resistance was applied on the subject’s fist. All normalization procedures were performed by the same examiner.

During execution of the PUP variants, subjects were instructed to position their hands on the force plate, aligning their index finger lateral to their acromion. While maintaining an angle of approximately 180° between the upper and lower body, subjects were asked to perform two variations of the PUP exercise (Figure 1), consisting of both a traditional PUP (supported on hands and feet) and modified PUP (supported on hands and knees). Subjects practiced these two conditions, attempting to lower and raise the body during task execution at a pace of four seconds per repetition (two seconds down, two seconds up). Subjects were asked to perform three continuous repetitions in each of the two conditions. During the execution of the push-up plus, the investigator made sure that the subject’s posterior thorax was rounded or curved at the end of the push phase. Subjects were afforded a rest period of at least one minute after each trial to minimize the effects of fatigue. The PUP conditions (modified vs. traditional) were randomized between subjects. A block of wood with a height of 10 cm was positioned in the middle of the force plate to standardized the depth of the eccentric phase of the PUP across subjects. The block was positioned so that the subjects would touch it by their sternum along their nipple line. The base of the hand and the index finger were marked with a tape on the force plate to ensure consistency with hand placement between trials.

### Statistical analysis

SPSS, version 20.0, was used for statistical analysis. Two-way repeated measures analysis of variance (ANOVA) were conducted to determine the effect of the exercise variant (traditional vs. modified) and elbow position (5° increments from 100° flexion to full extension) on the normalized EMG activity of each muscle of interest (upper trapezius, lower trapezius, serratus anterior, and infraspinatus) and on vGRF normalized to body weight (BW). Greenhouse-Geisser correction was implemented if Mauchly’s test revealed that the data violated the assumption of sphericity. Simple effects analyses were conducted for significant interaction effects using multivariate ANOVA. Bonferroni post-hoc procedures were conducted in the case of significant main effects. The criterion for statistical significance was set at the *P* = .05 level.

## Results

**Figure 2:**
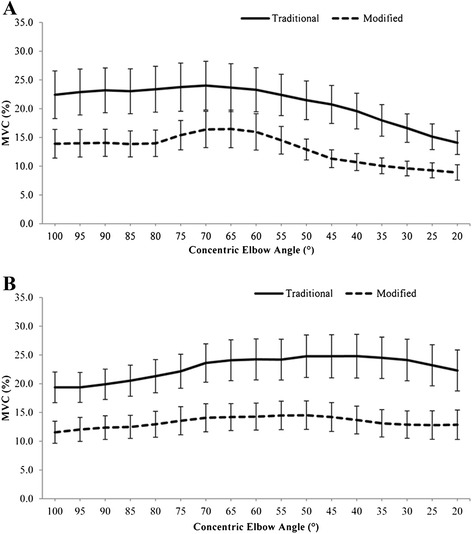
**EMG activity of the (A) Upper Trapezius and (B) Lower Trapezius during the concentric phase of the push-up plus variants.**

No significant interaction effect was found between exercise variant and elbow angle on UT EMG level (*F*[1.84, 38.53] = .97, *P* = .382) (Figure 2A). UT activation was significantly greater in the traditional versus the modified variant (*F*[1,21] = 17.15, *P* < .001), with a mean difference of 8%. UT activation was significantly affected by elbow angle (*F*[2.17, 45.65] = 7.81, *P* = .001), with a significant quadratic decrease in UT activation with elbow extension, as indicated by polynomial contrast (*F*[1,21] = 9.29, *P* = .006).

There was a significant exercise variant by elbow angle interaction effect on LT activation (*F*[2.64, 55.52] = 3.57, *P* = .024). Simple effects analyses revealed a significant effect of elbow angle on LT activation in both traditional (*F*[1,21] = 49.41, *P* < .001) and modified (*F*[1,21] = 34.30, *P* < .001) push-up variants. Graphical examination of the data (Figure 2B) indicates that LT activation increased sharply in both conditions until approximately 70° of elbow extension, with a more pronounced increase noted in the traditional condition. LT activation began to decrease again at approximately 50° and 40° elbow extension in the modified and traditional conditions, respectively. Simple main effects analyses also showed that traditional PUP had significantly higher LT EMG activity than modified PUP (*P* < .001) across all elbow positions, with a mean difference of 9.5%.

**Figure 3:**
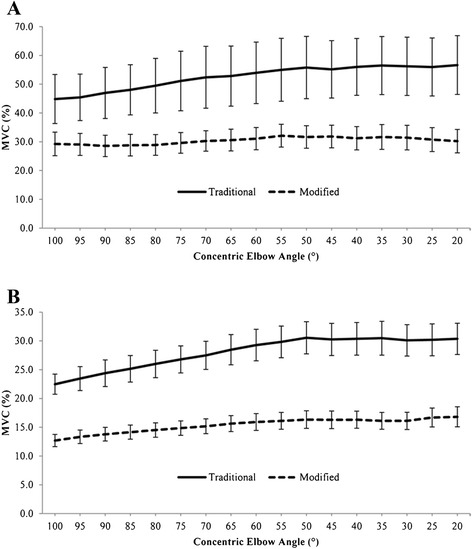
**EMG activity of the (A) Serratus Anterior and (B) Infraspinatus during the concentric phase of the push-up plus variants.**

There was a significant exercise variant by elbow angle interaction effect on SA activation (*F*[2.62, 54.93] = 3.52, *P* = .026). Simple effects analyses revealed a significant effect of elbow angle on SA activation in both traditional (*F*[1,21] = 28.59, *P* < .001) and modified (*F*[1,21] = 62.68, *P* < .001) push-up variants. Graphical examination of the data indicates that SA activation exhibited a greater increase across the elbow extension ROM in the traditional, compared to the modified, condition (Figure 3A).

There was a significant condition by elbow angle interaction effect on INF activation (*F*[2.69, 56.45] = 6.13, *P* = .002). Simple effects analyses revealed a significant effect of elbow angle on INF activation in both traditional (*F*[1,21] = 125.62, *P* < .001) and modified (*F*[1,21] = 130.08, *P* < .001) push-up conditions. Graphical examination of the data indicates that INF activation increased to a greater extent with elbow extension in the traditional condition, as compared to that in the modified condition. In addition, infraspinatus (INF) activation appeared to increase in both conditions until the elbow was extended to approximately 50°, after which, activation appeared to level off (Figure 3B). In addition, simple effects analyses also showed that traditional PUP exhibited significantly higher INF EMG activity than modified PUP (*P* < .001) across all elbow positions, with a mean difference of 12.6%.

**Figure 4:**
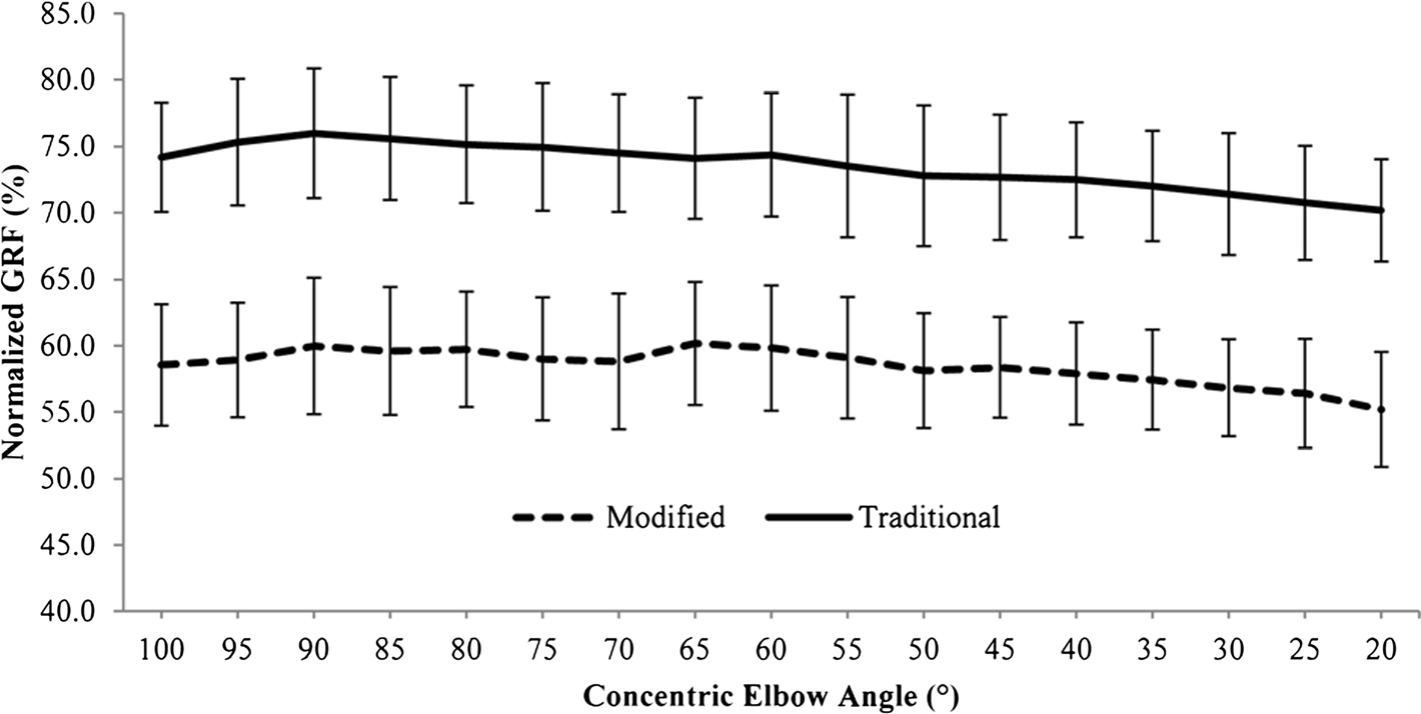
**Vertical ground reaction force normalized to subject’s weight during push-up plus variants.**

Vertical ground reaction force was normalized to the subject’s body weight. There was no significant interaction effect between the exercise variant and elbow position for the UT, (*F* [4.1, 85.4] = 1.34, *P* = .26) (Figure 4). vGRF was significantly affected by exercise variant, (*F* [1,21] = 52.15, *P* < .001) and elbow position, (*F* [3.2, 68.09] = 14.31, *P* < .001). vGRF was significantly higher during the traditional compared to modified PUP (*p* < 0.001), with a mean difference of 17%. vGRF was highest during the traditional PUP at 90° (76% BW) of elbow flexion and lowest at 20° (70% BW) of elbow flexion (*P* = .001). vGRF displayed a significant linear decrease across the ROM for both variants (*p* < .001).

## Discussion

The present study examined the effect of PUP variant and elbow position on EMG activity of four scapular stabilizer muscles and vertical ground reaction force during concentric contraction. To our knowledge, this is the first study that looked at EMG activity of shoulder muscle stabilizer and vGRF at various elbow position during push-up plus. The current data demonstrated that traditional PUP was characterized with greater EMG muscle activity in all the muscles tested during the entire elbow range of motion compared to modified PUP. In addition, the traditional PUP resulted in larger vGRF compared to modified PUP.

The push-up plus exercise is considered to be the best exercise to activate the Serratus Anterior [8,14]. It has been shown that SA EMG activity ranged from 69% to 120% maximum voluntary isometric contraction (MVIC) during some variants of push-up exercise [8,14,19,20,23,30,31]. In the current study, the SA EMG activity ranged from 45% to 57% MVC, which is below the value reported in the literature. The difference between the EMG values may be due to the fact that the current study performed the MVIC in a different method. However, in a recent study by Sciascia et al., they reported similar SA EMG activity to the present study during the push-up plus exercise [32]. Although, their results were from subjects with multi-directional instability and isolated anterior instability of the shoulder, they reported no significant differences between healthy and shoulder instability patients in SA EMG activity during the PUP exercise.

The present data also showed increased SA activity during elbow extension. During the modified PUP, there was only a 3% change in the entire concentric phase of the PUP. This shows that when performing the modified version of the PUP, the added motion of protracting the shoulder blade upon completion of the push-up repetition is not necessitated. The highest SA EMG activity during the concentric phase of the modified PUP happened at 55° of elbow extension, while in the traditional PUP, the highest SA EMG activity occurred at 20° of concentric elbow extension, which is during the plus phase of the exercise.

The UT and LT are considered to be two of the main scapular stabilizers [33]. Both the UT and LT contribute to scapular upward rotation during humeral elevation [34]. However, the UT also contributes to anterior tilting by drawing the clavicle medially and upwards [34], which could decrease the subacromial space and can increase the risk of subacromial impingement [5]. The LT also assists in posterior tilting during humeral elevation [35]. Thus, the goal in most shoulder impingement rehabilitation protocol is to decrease the activity of the UT compared to the LT during arm elevation. Consequently, the scapula will be in a more posteriorly tilted position, which increases the subacromial space. In the current study, both variants of PUP started with the UT (24% MVC) having an increased muscle activity compared to the LT (19% MVC). However, during the mid-range of the concentric phase of the PUP, there was a shift in activation pattern. The UT (20% MVC) decreased and the LT (24.8% MVC) increased its EMG activation starting at 70° of elbow extension up to the plus phase (i.e. 20° of elbow extension) of the traditional PUP exercise. These results are in accordance with previous studies that examined different variance of push-up plus exercise with the UT ranging from 20.5% - 25.2% MVC [15,31]. For the LT, Park et al. [15] examined the LT during wall push-up plus and wall slide device and reported a range of 11.6% - 16.1% MVC [15]. The current study had a higher LT EMG activity. This difference can be attributed to the position that the push-up plus was implemented. The PUP exercise performed in Park et al. [15] study was accomplished standing up against the wall while the current study had the subjects complete the PUP on the ground. The wall PUP is considered to be less demanding than the traditional on the ground PUP [8]. The modified PUP did not change muscle activation patterns until the elbow was at 55° of elbow extension. The shift of muscle activity into a more LT than UT during the PUP is important to avoid impingement of soft tissues under the subacromial space.

The infraspinatus is one of the rotator cuff muscles. Its main function is to externally rotate the humerus [36]. It also serves to assist in centralizing the humeral head into the glenoid during shoulder motion [37]. In the current study, the infraspinatus was activated during both PUP variants. The traditional PUP had the greatest EMG activity (30% MVC) when the elbow was less than 50° of elbow flexion. This result is in accordance with Sciascia et al. [32] while subjects performed the PUP exercise. During humeral elevation in the scapular plane, infraspinatus activity has been reported to be between 10% - 25% MVC [29,38]. Conversely, it has been shown that sidelying external rotation exercise and prone external rotation can elicit a great amount of EMG activity equal to 62% and 63% MVIC, respectively [32,39]. However, these exercises were designed to isolate the INF. The result of the present study demonstrates that PUP exercise is not an ideal exercise to strengthen the INF. Even with the increased demand or complexity to maintain stability in the shoulder musculature during the PUP exercise, the INF had the same amount of activation with scapular plane elevation.

Our results indicated greater vGRF across the ROM in the traditional versus the modified PUP variant. This finding confirms those of previous studies examining ground reaction forces in these variants in both static positions [16] and dynamic movement [25,40]. In the traditional variant, vGRF ranged from 70.18% - 75.99% body weight, while in the modified variant, vGRF ranged from 52.95% - 57.95%. These ranges compare well to the percentage of body weight supported in the “up” and “down” positions of the traditional and modified variants reported by Suprak, Dawes, and Stephenson [16]. Ebben et al. reported similar findings with respect to the peak GRF in various push-up exercises, which included traditional and modified variants, as well as those with feet elevated, and those with hands elevated on boxes of increasing height [40]. These investigators reported increasing peak vGRFs as the push-up variant was altered from hands elevated 60.96 cm to modified to hands elevated 30.48 cm to traditional to feet elevated 30.48 cm and 60.98 cm. These results are in support of the present data in that they confirm the pattern of greater vGRF in the traditional versus the modified push-up. Gouvali and Boudolos also reported greater vGRF in the traditional variant, as compared to the modified [25]. However, they reported peak vGRFs of 66% and 53% in the traditional and modified push-up ROM, respectively. This difference between their findings and those in the present study may be related to the different subjects included in the two studies. In their study, Gouvali and Boudolos included only male subjects, while the present study included both males and females. This difference may have impacted the distribution of body mass in subjects in the two studies, leading to a greater percentage observed in the present data [25].

The second important finding with regards to the vGRF in this study was the significant linear decrease in vGRF with elbow extension in the concentric portion of both variants. We hypothesize that the increase in vGRF with elbow extension is the result of the whole-body center of mass location moving further from the point of contact (feet or knees) with the support surface (floor) in the horizontal direction, resulting in greater gravitational torque that must be overcome by the vGRF in order to perform the exercise. This finding, again, supports those of Suprak, Dawes, and Stephenson, who reported greater vGRF in the “down” versus the “up” position of both variants [16].

Lastly, one of the more surprising finding in the current study was that the subjects did not lock their elbows at the end of the concentric phase of the push-up plus. The minimum elbow extension angle was 20° during the concentric phase of the PUP exercise. The investigators made sure that all the subjects were performing the exercises correctly, and they were protracting their shoulders at the end of the concentric phase of the PUP. To our knowledge, this is the first study that examined the entire elbow angle range of motion during the concentric phase of the PUP. The usual direction to subject performing the PUP is to extend their elbow to a standard push-up position and continued rise up by protracting the scapula [14]. There are no studies that reported the specific elbow angle position throughout the entire concentric phase of the push-up plus exercise.

There were limitations that needed to be acknowledged and addressed regarding the present study. The first limitation concerns the elbow kinematics in 2D during the PUP. In order to avoid error in projection angle of the elbow, the investigator made sure that the camera was directly perpendicular to the elbow motion. Additionally, the hand placement of each subject was clearly marked on the force plate to maintain consistent subject location between trials. The second limitation is EMG cross talk between adjacent muscles. This limitation is inherent in every surface EMG study. In order to address this limitation, the investigator did a specific manual muscle test for every muscle tested before the normalization of the signal and during each trial.

## Conclusion

This current study demonstrated that traditional PUP resulted in increased EMG activity of the LT, SA, UT and INF compared to the modified PUP during the concentric phase. The highest activity of the serratus anterior occurred at 55° of elbow extension during the concentric phase of the traditional PUP. This suggests that the plus phase of the PUP is not necessary to attain greater activity of the SA when performing traditional PUP. However, during the modified PUP, the highest activity of the SA occurred at the plus-phase. Additionally, the traditional PUP resulted in increased vGRF in the entire elbow ROM during the concentric phase of the exercise compared to modified PUP. These results can be helpful in clinical application when prescribing PUP exercises to patients with shoulder pathology (i.e. subacromial impingement syndrome). However, care should be taken when considering these exercises for patient population, since the current study utilized healthy subjects. It should be noted that progression is important when recommending PUP variants. It is beneficial to start with the modified PUP variant earlier in the shoulder rehabilitation phase since it demands lesser internal torque in the upper extremity.
